# Effects of Market Incentives and Livelihood Dependence on Farmers’ Multi-Stage Pesticide Application Behavior—A Case Study of Four Provinces in China

**DOI:** 10.3390/ijerph19159431

**Published:** 2022-08-01

**Authors:** Xiuling Ding, Apurbo Sarkar, Lipeng Li, Hua Li, Qian Lu

**Affiliations:** 1 College of Economics and Management, Northwest A&F University, Xianyang 712100, China; dxl1227@nwafu.edu.cn (X.D.); apurbo@nwafu.edu.cn (A.S.); lihua7485@nwafu.edu.cn (H.L.); 2 School of Economics and Management, Ningxia University, Yinchuan 750021, China; feixiang2022@nxu.edu.cn

**Keywords:** market incentives, livelihood, application, farmers’ behavior, pesticide, probit model

## Abstract

Improvement in pesticide application and efficiency structure has long been recognized as having great significance in reducing pollution, ensuring food safety, and promoting green agricultural development. Based on theoretical analysis, using the survey data of 766 farmers in key tea areas in Shaanxi, Sichuan, Zhejiang, and Anhui provinces in China, the study empirically analyzes the influence of market incentives and livelihood dependence on farmers’ multi-stage pesticide application behavior. More specifically, the study employed ordered probit analysis to craft its findings. The dependent variable of this study is the multi-stage pesticide application problem of farmers, and the core independent variables are market incentives and livelihood dependence, and the judgment is based on the core variable coefficients of the econometric model of farmers at each stage. The study found the following: (i) Market incentives significantly prompted some farmers to give up synthetic pesticide application and farmers tend to choose green pesticides in the type of pesticide application. (ii) Livelihood dependence meant that the proportion of tea income significantly prompts farmers to apply pesticides, and also creates a tendency for farmers to choose green and low-toxic pesticides in the type of pesticide application. The planting period tends to have a moderate impact on applying green and low-toxic pesticides. (iii) The interaction term of market incentives and the proportion of tea income has no significant impact on farmers’ multi-stage pesticide application behavior. The interaction term of market incentives and planting years has impacted negatively on whether farmers apply pesticides, and has no significant impact on farmers’ choice of pesticide application types, but makes farmers increase the amount of green and low-toxic pesticides. (iv) The education level of the household head significantly promotes farmers to choose green and low-toxic pesticides. Seemingly, the brand effect of pesticides significantly encourages farmers to choose green and low-toxic pesticides. In external support, technical training significantly encourages farmers to choose green and low-toxic pesticides. Furthermore, better infrastructure and local market conditions significantly encourage farmers to reduce the use of conventional pesticides.

## 1. Introduction

Ever-increasing trends in the population and demands for food and fiber foster the chemical interactions in agriculture. However, most developing countries rely substantially on intensifying production, which eventually lowers the land quality and reduces the resistance to pests and other diseases [1,2,3]. Pesticides are known as substances that are frequently employed in agriculture to eliminate bugs, parasites, and weeds; however, the widespread use of chemical-based pesticides has long been recognized as a major threat to global biodiversity, public health, and environmental safety, and eventually hamper the ecosystem and sustainability [4,5]. Due to its severe consequences, both in environmental and human life, global communities and governments are trying to limit the usage of synthetic pesticides and employing several policies to encourage efficient usage of pesticides [6,7,8]. China is the world’s largest consumer of pesticides, but the effective utilization rate of pesticides is less than 40% [9,10]. The pollution caused by excessive application of pesticides and high residues is distributed in the vast rural areas, which seriously threatens food safety and the environment of production areas [11,12]. As the main body of the direct application, farmers’ pesticide reduction and application structure are extremely severe, and the use rate and application intensity of conventional pesticides remain high [13,14]. On the contrary, the control coverage rate of green and low-toxic pesticides is low, but their unit investment far exceeds the optimal level [15,16]. Promoting farmers’ pesticide reduction and application structure optimization has become a key issue to reduce pesticide pollution, ensure food safety, and promote the green development of agriculture.

With the improvement in the market system, technological progress, and consumption upgrade, the impact of market incentives on farmers’ production behavior has become increasingly prominent [10,17,18]. Market incentives are a combination of price signals formulated by market organizations and product quality grades [19]. They are quality differential pricing standards implemented by market organizations based on traditional product appearance and texture, which have an important impact on farmers’ pesticide application behavior [20,21,22]. Relevant studies have found that the expected external income significantly affects the quality and safety control behavior of vegetable farmers [19,23] and the effect of pesticides on farmers’ cognition of potential income is an important factor affecting their pesticide usage [24,25]. According to the theory of behavior, farmers’ pesticide application behavior is not only closely related to external driving forces such as market incentives but also closely related to farmers’ livelihood dependence [26,27]. Seemingly, livelihood dependence refers to the degree of dependence of farmers’ production and life on agriculture [28]. In particular, under external intervention, rational farmers will generally adjust their pesticide application behaviors according to their livelihoods and subsistence dependence is generally reflected through income. For example, Pan et al. [29] found that farmers who pay more attention to income are less likely to have environmentally friendly pesticide application behavior. In a study on pesticide usage behavior among Kuwaitian farmers, Jallow et al. [30] found that farmers will reduce agricultural labor input for field management and increase the amount of pesticide application after part-time employment. In a study in Barbados, Yawson [31] found that concurrent employment promotes the production behavior of “resilient systems” for farmers through the income effect and substitution effect.

The existing results provide a good reference for the study, but it can still be expanded on in the following two ways: First, though research on farmers’ pesticide application behavior based on market incentives or livelihood dependence has gradually increased, there is a lack of studies that integrate these two major factors in an integrated framework. Moreover, the regulatory role of livelihood dependence on market incentives and farmers’ pesticide application behavior has not been paid much attention by the existing studies. Second, the existing articles only study whether farmers apply pesticides, the types of pesticides or the number of pesticides applied, and the multi-stage pesticide application behavior in the decision-making process of whether farmers apply pesticides, selection of pesticide types, and selection of pesticides have not been clarified yet. Given this, the study divides the pesticide application behavior of farmers into multiple stages of whether to apply pesticide, selection of pesticide type, and selection of pesticide amount. The impact of market incentives and livelihood dependence on farmers’ pesticide application behavior was comprehensively analyzed, to provide a decision-making reference for the government to formulate relevant policies to promote farmers’ pesticide reduction, structure optimization, and agricultural green transformation.

## 2. Theoretical Analysis and Hypotheses

### 2.1. The Impact of Market Incentives on Farmers’ Multi-Stage Pesticide Application Behavior

Existing studies (such as Sarkar et al. [17], Yang et al. [32], and Liu and Wu [33]) generally emphasize the importance of market organization in the green transformation of agriculture, especially in realizing the organic connection between “small farmers’ production” and “big market demand”. However, a growing number of studies have established that a farmer’s household has a close interest and linkage mechanism with farmers, which plays an important role in the pesticide application behavior of farmers [34,35]. Seemingly, market incentives, as a combination of price signals and product quality levels, are the most effective tools and means of market organization, and will inevitably affect farmers’ pesticide application behavior [36,37]. Market incentives are a hierarchical purchasing system adopted by market organizations to ensure the quality of agricultural products and gain competitive advantages. The mechanism of action is as follows: First, the application of pesticides will affect the texture, taste, and quality of agricultural products and other transaction characteristics, and this is more obvious in high-value tea. The market organization can identify and differentiate the quality of tea by technical means and purchase price [38]. Second, with the acceleration of the marketization process, farmers are transitioning from a subsistence small-scale peasant economy to a commodity economy. To successfully enter the market and obtain maximum benefits, farmers must organize production according to the product quality preferences transmitted by the market organization [39]. Third, to meet the demands of their development and consumption pattern, market organizations require farmers to organize the production and application of pesticides following standards and use the information and technical methods to conduct pesticide testing on farmers’ products, which eventually fosters farmers to optimize their pesticide application behavior [40]. Because of this, this study proposes the first hypothesis.

**Hypothesis** **1** **(H1).** *Market incentives have a significant impact on farmers’ multi-stage pesticide application behavior.*

### 2.2. The Impact of Livelihood Dependence on Farmers’ Multi-Stage Pesticide Application Behavior

For intensifying agricultural production, the impact of livelihood dependence on farmers’ behavioral choices is generally considered from the perspective of household income and consumption [41,42]. The importance of income will affect the factor input of farmers and their energy input and the higher the proportion of product income, the more farmers need to consider potential market risks, and generally arrange their factor inputs according to the corresponding market requirements [43,44]. However, farmers also have speculative preferences, which may be due to maximizing short-term returns and being willing to take risks that violate market institutional arrangements. Farmers with more household consumption tend to pay more attention to product quality and safety, will control factor inputs reasonably and safely [45], and even adopt two different factor inputs and management methods for the same crop [46]. Compared with intensifying agricultural production, tea has its particularity, and household consumption of farmers only accounts for a small part of the total output, but livelihood dependence can still affect farmers’ pesticide application behavior through two aspects. The first is income; due to differences in the contribution of agricultural livelihoods to household income, farmers will make decisions on pesticide applications according to their importance and eventually maximize the total household income within a production cycle [47,48]. Farmers have limited economic rationality when farmers prefer risk aversion; tea income is relatively important to them so they will arrange spraying activities according to general market standards, and when farmers prefer risk speculation, tea income is relatively important to them [49,50]. Therefore, the goal of maximizing economic benefits is to arrange efficient pesticide management activities. 

The second is the planting period. Farmers will accumulate experience and experience in the process of tea planting and become an important component of their human capital and family culture [51] and preventing farmers from switching to other livelihood models [52]. However, the fixed production model formed by the experience attached to the planting years affects farmers’ pesticide application behavior [53,54]. Generally, the longer the planting years, the more experienced the farmers are and the more knowledgeable they are about the drug use and growth habits of tea [55,56]. However, the farmers who have been planting for a long time are indifferent to or even reject new pesticides or new technologies, and are highly dependent on pesticides [57]. Given this aspect, this study proposes the second hypothesis.

**Hypothesis** **2** **(H2).** *Subsistence dependence has a significant impact on farmers’ multi-stage pesticide application behavior.*

### 2.3. The Impact of the Interaction between Market Incentives and Livelihood Dependence on Farmers’ Multi-Stage Pesticide Application Behavior

As discussed above, market incentives affect farmers’ multi-stage pesticide application behavior through a series of mechanisms, and livelihood dependence may moderate the impact of market incentives on farmers’ multi-stage pesticide application behavior. First, in the face of production pressure, technical pressure, and quality pressure under market incentives, income dependence has an important impact on farmers’ production choices [58,59]. On the one hand, farmers may follow market incentives to ensure a stable income and choose to use pesticides reasonably [60,61]. On the other hand, they may also use market incentives to speculate to grab more economic benefits, which may lead to the failure of the market incentives or even counterproductive effects [62,63]. Second, market incentives have formulated more detailed product quality requirements and production behavior standards for farmers. The longer the planting years, the more the farmers have the corresponding technical capabilities and can better meet the needs of market organizations [64,65]. Therefore, it can be said that the planting years can strengthen market incentives and affects farmers’ pesticide application behavior. However, it should be noted that the longer the planting period, the more likely farmers will conflict with market incentives under the guidance of experience and the solidified chemical production model, and some farmers can even rely on planting experience to “work around” without optimizing pesticide use to obtain higher rates. Therefore, the planting years will send a market signal of “traditional high-quality agro product”, which will weaken the effectiveness of market incentives and cause the market incentive mechanism to fail [65,66]. Another possibility is that the planting period will send a market signal of “traditional high-quality tea”, which will weaken the effectiveness of market incentives and cause the market incentive mechanism to fail. Based on this, the study proposes the third hypothesis.

**Hypothesis** **3** **(H3).** *Subsistence dependence has a moderating effect between market incentives and farmers’ multi-stage pesticide application behavior.*

## 3. Materials and Methods

### 3.1. Data Sources

The data in this study come from the research group’s survey data of farmers in 8 key tea areas in Shaanxi, Sichuan, Zhejiang, and Anhui from July to September 2021. First, the research team selected important tea-producing areas in Zhejiang, Sichuan, Shaanxi, and Anhui provinces for research based on tea production and economic development. Next, we randomly selected 3 townships from each county; after that, 2 administrative villages were randomly selected from each township. Finally, 10 to 20 farmers were selected by random sampling in the village. The survey was mainly conducted by questionnaire survey combined with interviews and obtained information such as farmers’ pesticide application behavior, household head characteristics, basic family situation, and organizational support. After excluding invalid questionnaires, a total of 766 valid questionnaires were obtained. 

### 3.2. Model Construction

The study employed ordered probit analysis to test the model, which is widely used by similar studies (such as Oyetunde-Usman et al. [67], Maguza-Tembo et al. [68], and Pilarova et al. [69]). In statistics, ordered probit is a generalization of the widely used probit analysis to the case of more than two outcomes of an ordinal dependent variable [70,71]; therefore, the ordered probit is the best option for the study. 

#### 3.2.1. Farmers’ Decisions of Pesticide Application

To estimate the impact of market incentives and livelihood depends on whether farmers apply pesticides. The following probit model was constructed:

(1) $$W_{i}=\alpha K_{i}+\beta H_{i}+\gamma K_{i}*H_{i}+\eta Z_{i}+\mu_{i}\;$$

In Formula (1), it $W_{i}$ represents whether farmers apply pesticides; it $K_{i}$ represents livelihood dependence, $H_{i}$ represents market incentives, $K_{i}*H_{i}$ represents the interaction term between market incentives and livelihood dependence, $Z_{i1}$ is a control variable, α, β, γ, η are coefficients to be estimated, and $\mu_{i}$ are residual terms, subject to *N* (0,1) distribution.

#### 3.2.2. Farmers Choose the Type of Pesticide Application

To estimate the impact of market incentives and livelihood dependence on farmers’ choice of pesticide application types, the following probit model was constructed:

(2) $$WD_{i}=\chi K_{i}+\eta H_{i}+\gamma K_{i}*H_{i}+\omega Z_{i}+\nu_{i}\;$$

In Formula (2), it $WD_{i}$ represents the choice of pesticide application type by farmers, which χ,η,γ,ω is the coefficient to be estimated, and $\nu_{i}$ the residual item, which obeys the *N* (0,1) distribution.

#### 3.2.3. Farmers’ Choice of Pesticide Application Rate

To estimate the impact of market incentives, livelihood dependence, etc. on farmers’ choice of pesticide application rate, an ordered probit model for farmers’ selection of pesticide application rate was constructed. The equation is shown in Equation (3):

(3) $$\left\{\begin{array}{c} WL_{i}=\phi_{0}K_{i}+\varphi_{0}H_{i}+\xi_{0}K_{i}*H_{i}+\zeta_{0}Z_{i}+\sigma_{i0}\;,\mathrm{if}WD_{i}=0\\ WL_{i}=\phi_{1}K_{i}+\varphi_{1}H_{i}+\xi_{1}K_{i}*H_{i}+\zeta_{1}Z_{i}+\sigma_{i1}\;,\mathrm{if}WD_{i}=1 \end{array}\right.$$

In Formula (3), it $WL_{i}$ is the selection of the amount of pesticide applied by the farmers; the selection of the $WD_{i}$ type of pesticide applied by the farmers ϕ, φ, ξ, ζ is the coefficient to be estimated and $\sigma_{i}$ is the residual term.

### 3.3. Variables and Descriptive Statistics

#### 3.3.1. Farmers’ Multi-Stage Pesticide Application Behavior

Three variables were used to characterize the multi-stage pesticide application behavior of farmers. To ensure the validity of the data, after the investigators asked farmers whether to apply pesticides, they ensured that the farmers determine the type of pesticide application and the amount of pesticide application in the form of explanations. As shown in Table 1, in terms of whether to apply pesticides, 644 households apply pesticides, accounting for 84.07% of the total sample. Whereas, a total of 122 households do not apply pesticides, accounting for 15.93% of the total samples. In the selection of application types, green and low toxicity are selected. There are 431 pesticide farmers, accounting for 66.93% of the sample of pesticide farmers and 213 farmers choose conventional pesticides, accounting for 33.07% of the sample of pesticide farmers. However, in terms of pesticide application rate, 18.31% of farmers use less conventional pesticides, and 42.25% of farmers use green fertilizer. The amount of conventional pesticides has not changed, and 39.44% of the farmers have increased the number of conventional pesticides. While 25.06% of the farmers reduced the amount of green and low-toxic pesticides, 34.34% of the farmers have the same amount of green and low-toxic pesticides, and 40.60% of the farmers have increased the amount of green and low-toxic pesticides.

#### 3.3.2. Core Independent Variables

Market incentives, as a combination of price signals and product quality grades, have an important impact on farmers’ pesticide application behavior [72,73]. Therefore, the market incentive is set as a key variable (whether the market organization has differentiated pricing of products with different drugs due to the importance of pollution-free, green, and organic products; Yes = 1; no = 0). Among the sample farmers, 30.55% of the farmers believed that market organizations would price differentially on products with different medicines because of the importance of pollution-free, green, and organic products. At the same time, the study found that 69.45% of market organizations would not make difference in the use of medicines, because of the importance of pollution-free, green, and organic products. Livelihood dependence reflects the contribution of agricultural production to farmers’ households. Based on the existing research (such as Van Ho et al. [74] and Zheng et al. [51]) and the characteristics of the tea industry, the two variables of tea income proportion and planting years are used to characterize the livelihood dependence. In the sample, 43.86% of farmers have more than 20% of their income from tea, and 15.93% of farmers have more than 50% of their income from tea; farmers with 10 years or more of tea cultivation years account for more than 90% of the total sample.

#### 3.3.3. Control Variables

Farmers’ multi-stage pesticide application behavior is closely related to farmers’ interpersonal characteristics, family characteristics, land endowment, and affect cognition. Referring to the relevant literature (for example Mubushar et al. [75], Lekei et al. [76], and Istriningsih et al. [77]), the study selected the characteristics of the household head (age of household head, education level of household head), family characteristics (number of labor force, household income level), garden endowment (tea garden area, tea garden altitude, road condition), pesticide cognition, yield effect, pesticide brand effect, pesticide environmental effect), external support (technical training), and regional characteristics were used as control variables to ensure the estimated effect.

## 4. Results

### 4.1. Empirical Results and Analysis of whether Farmers Apply Pesticides

The probit model was used to verify the influence of market incentives and livelihood depending on whether farmers apply pesticides. As shown in Table 2, the model passed the 1% significance test, and the overall fitting effect is good. The specific results and analysis are as follows.

#### 4.1.1. Core Variables

Market incentives significantly promote farmers to give up pesticide application, that is, farmers who obtain higher market incentives will give up pesticide application [73]. With the improvement in the agricultural product commodity market and industrial chain, market organizations have more effects to convey interactions with farmers, while farmers are in a dominant position within this aspect, and they must meet the needs of market organizations. For cooperation and development, it is necessary to formulate effective market incentives according to the local context to encourage farmers to give up pesticide applications [78,79]. (2) In livelihood dependence, the proportion of tea income significantly promotes the application of pesticides by farmers, and the farmers with a higher proportion of tea income are more likely to apply pesticides. Farmers are rational people and have strong risk aversion preferences [80,81]. To reduce the loss of crop diseases and insect pests in order to obtain a stable income, farmers tend to use pesticides. Planting years had a positive effect on whether farmers applied pesticides but did not pass the significance test [64]. (3) The interaction term between market incentives and planting years encourages farmers to apply pesticides at the 5% significance level [82]. In a study on pesticide reduction motivation among UK farmers, Hillocks [83] highlighted that “the longer the planting years, the better the farmers understand the growth habits of tea and market trading rules, and can rely on their own experience to respond to market incentives”. However, in developing countries, especially in China, there are several market signals such as the longer the planting years “the better the tea quality” and better control experience which will weaken the incentives for market organizations. It can be seen from the coefficients of the measurement model in Table 2 that the interaction coefficient between market incentives and planting years is positive, and it has passed the 5% significance test. Therefore, it is concluded that the interaction term between market incentives and planting years can encourage farmers to apply pesticides. This conclusion conforms to the logic of econometrics. Therefore, under the adjustment of planting years, the effect of market incentives on farmers’ pesticide application behavior is weakened. Based on these factors, the study selected market incentives and subsistence dependence (income, and planting years) as core variables. 

#### 4.1.2. Control Variables

Based on the assumption of the existing studies (such as Lekei et al. [76], Ngowi et al. [84], and Mengistie et al. [85]) the study selected the head of household characteristics, family characteristics, field endowment, cognition, external support as control variables. Among the characteristics of household heads, the education level of household heads significantly promoted the application of pesticides by farmers. The educational level of farmers is closely related to the degree of concurrent employment, and the higher the degree of concurrent employment, the more inclined they are to use pesticides to replace labor. Among the garden land endowments, the altitude of the tea garden significantly promoted farmers to give up pesticide application. The frequency and damage of tea pests and diseases are closely related to altitude. The higher the altitude, the lower the temperature and the less active pests and diseases, so farmers can give up the application of pesticides. Among the cognitive characteristics, the effect of pesticide yield significantly encourages farmers to apply pesticides, and the effect of pesticide brands significantly encourages farmers to give up pesticides. Most farmers are risk-averse farmers and tend to use pesticides to reduce yield losses; branding is very important for farmers’ product sales and selling prices. When farmers realize that the use of pesticides will affect the brand of the place of origin, farmers will give up the application to ensure sales and selling prices. 

### 4.2. Empirical Results and Analysis of Farmers’ Choice of Pesticide Application Types

As shown in Table 3, the model passed the 1% significance test, and the overall fitting effect was good. The specific analysis is as follows.

#### 4.2.1. Core Variables

Market incentives have significantly prompted farmers to choose conventional pesticides. Due to the incomplete technical support of market incentives and insufficient market incentives, it is easy for the tea market to form a collective mechanism that leads farmers to choose cheap and effective conventional pesticides. In livelihood dependence, the proportion of tea income significantly encourages farmers to choose green and low-toxic pesticides. Most farmers are risk-averse, and the higher the income proportion, the farmers tend to choose green and low-toxic pesticides to ensure the quality of tea and obtain stable income. The planting age significantly prompts farmers to choose conventional pesticides. The longer the age of tea planting in tea gardens, the accumulation of farmers’ experience, the solidification of chemical production models, and the enhancement of pest and disease resistance in old tea gardens make farmers highly dependent on conventional pesticides with stronger toxicity. The interaction term between market incentives and the proportion of tea income and planting years has no significant effect on the type of pesticide application by farmers.

#### 4.2.2. Control Variables

Among the characteristics of a household head, the education level of the household head significantly encourages farmers to choose green and low-toxic pesticides. Education level is closely related to information acquisition and personal ability. Farmers with high education levels generally have a richer knowledge and higher awareness of green and low-toxic pesticides and tend to choose green and low-toxic pesticides. In effect cognition, pesticide yield effect, and pesticide brand effect significantly prompted farmers to choose green and low-toxic pesticides. Farmers are a micro-subject in both economy and society. The more objective their cognition of the yield effect and brand effect of pesticides, the better they can distinguish the overall pros and cons of conventional pesticides and green and low-toxic pesticides. The brand effect of pesticides significantly encourages farmers to choose green and low-toxic pesticides. In external support, technical training has significantly promoted farmers to choose green and low-toxic pesticides. Technical training is an important source of information and technology for farmers, which can promote farmers to understand green and low-toxic pesticides and improve their technical literacy, thus encouraging farmers to choose green and low-toxic pesticides.

### 4.3. Empirical Results and Analysis of Farmers’ Choice of Pesticide Application Rate

As shown in Table 4, the model passed the 1% significance test, and the overall fitting effect was good. The specific analysis is as follows.

#### 4.3.1. Farmers’ Choice of Conventional Pesticide Dosage

The planting years significantly encourage farmers to increase the use of conventional pesticides. This is because, on the one hand, the longer the planting period, the stronger the resistance to pests and diseases in the tea garden, and the decreased ability of conventional pesticides to prevent and control pests and diseases, which leads to the “easy increase but difficult to decrease” in the number of conventional pesticides used by farmers. The production concept and chemical production model formed during the popularization of chemical technology is highly dependent on the dosage of conventional pesticides, prompting farmers to increase the dosage of conventional pesticides. Market incentives and the proportion of tea income have no significant impact on farmers’ conventional pesticide usage. 

Among the garden land endowments, the area of tea gardens and road conditions significantly encouraged farmers to reduce the use of conventional pesticides. With the expansion of the operation scale, on the one hand, the factor allocation of farmers shows economies of scale, and the overall level of pesticide application is better than that of small-scale farmers. In terms of conventional pesticide dosage, road conditions are closely related to the difficulty in pesticide application by farmers. Due to road conditions, it is difficult to transport equipment and water for pesticide application to tea gardens, thus significantly encouraging farmers to reduce the number of conventional pesticides. In pesticide cognition, the effect of pesticide yield significantly encourages farmers to increase the number of conventional pesticides, that is, when farmers link production to pesticides, it will lead to excessive application of conventional pesticides; the effect of pesticide brand significantly encourages farmers to reduce the number of conventional pesticides, and the tea market is affected by the brand of origin, farmers who choose conventional pesticides are increasingly aware of the importance of good origin brands to tea sales, which will reduce the number of conventional pesticides. Technical training has significantly facilitated farmers to reduce the use of conventional pesticides. Technical training can significantly increase farmers’ knowledge of pesticides and encourage farmers to adopt more scientific means to control pests and diseases, so farmers who have received technical training can reduce the number of conventional pesticides.

#### 4.3.2. Farmers Choose the Dosage of Green and Low-Toxic Pesticides

Market incentives have significantly encourage farmers to reduce the use of green and low-toxic pesticides. To meet the upgrading of consumer demand, the market organization adopts the pricing standard of high quality and high price, and farmers will reduce the amount of green and low-toxic pesticides to obtain an higher income. The interaction between market incentives and planting time significantly encouraged farmers to increase the amount of green and low-toxic pesticides. Farmers who choose green and low-toxic pesticides have a high awareness of product quality. On the premise of a rich planting experience, farmers can add a certain amount of green and low-toxic pesticides according to market incentives. (2) Control variables. Among household characteristics, the level of household income significantly encourages farmers to increase the amount of green and low-toxic pesticides. With the increase in household income level, farmers can afford the cost of green and low-toxic pesticides and tend to use green and low-toxic pesticides to replace labor. Among the garden land endowments, the area of tea gardens significantly encourages farmers to reduce the use of green and low-toxic pesticides. With the increase of scale, on the one hand, the factor allocation of farmers who choose green and low-toxic pesticides presents economies of scale, and the overall pesticide application level is better than that of small-scale farmers; Substitute, thereby reducing the application rate of green low-toxic pesticides. In pesticide cognition, the effect of pesticide brands significantly encourages farmers to reduce the amount of green and low-toxic pesticides. Tea sales are greatly affected by the brand of the place of origin. Therefore, farmers who choose low-toxicity will reduce the amount of green and low-toxic pesticides when they realize the negative effects of pesticides on the brand of the place of origin.

### 4.4. Robustness Test

The robustness test was carried out based on Model 1–Model 8. Specifically, the logit model was used to replace the probit model of Model 1–Model 4. The logit model replaces the ordered probit model of Model 4–Model 8, thereby obtaining Model 9–Model 16. It can be seen from Table 5 that after replacing the model, the estimated coefficients of market incentives and livelihood dependence on farmers’ multi-stage pesticide application behavior are consistent with the previous model results, indicating that the model results are relatively robust.

## 5. Discussions

Promoting the reduction in pesticide use for farmers and the optimization of the application structure are the key issues for reducing pesticide pollution, ensuring food safety, and promoting the green development of agriculture [86]. The notion brings the prospects of green pest management. In China, green and low-toxicity pesticides will be indicated on the pesticide label [73]. Internationally, these components are usually ranked at various levels, such as the green level which means the pesticide is safe and the blue label which means a pesticide has low-toxicity [87]. Green or low-toxicity pesticides are generally made from biological sources, tweaking natural hormones from plant sources, or low-hazard chemicals, either singly or mixed [88,89]. Typical green and low-toxic pesticides include Liuyangmycin, Abamectin, Methoxyabamectin, Mancozeb, Oxyfluorfen, and glyphosate. The prominent feature of green and low-toxic pesticides is that they are less harmful to human health and the environment, and can better promote the common development of human beings and nature [90].

However, in previous studies, scholars have mainly studied pesticide application by farmers from the aspects of policy incentives [91,92], informal systems [93,94,95], organization and service outsourcing [96,97], and household resource endowments [98,99]. However, most of them explored those aspects in an isolated way and do not present any comprehensive model to test the impact of market incentives and livelihood dependence on farmers’ pesticide application behavior in an integrated manner. The research innovatively proposes the decision-making process of farmers’ multi-stage pesticide application behavior and divides farmers’ pesticide application behavior into three stages by exploring: (i) whether to apply pesticide, selection of pesticide type, and intensity, (ii) constructing a research framework on market incentives, livelihood dependence, and farmers’ pesticide application behavior, and revealing farmers’ multi-stage pesticide application behavior decision-making mechanism, and (iii) expanding the research framework of farmers’ behavior in decision-making, and providing a reference for related scholars’ research and government policy formulation. 

The results show that market incentives are an important external driving force affecting farmers’ pesticide application behavior, while subsistence dependence is an important internal force affecting farmers’ pesticide application behavior. In the review study on integrated pest management practices in Europe, Lefebvre et al. [91] found similar findings and confirmed that incentive-based instruments impact profitability and drive farmers to adopt innovation in pesticide management. However, market incentives, livelihood dependence, and their interaction terms did not play a role in the multi-stage pesticide application behavior of farmers (including whether farmers applied pesticides, selection of pesticide types, and selection of pesticide rates). This leaves Hypothesis (H1), Hypothesis 2 (H2), and Hypothesis 3 (H3) only partially verified. The reason for this result is that in the process of farmers’ multi-stage pesticide application behavior, farmers have various types and their pursuit of interests, which changes the mechanism of market incentives and livelihood dependence. Therefore, market incentives and livelihood dependence have different results in the multi-stage pesticide application behavior of farmers. In a study in Yunnan province, China, Udimal et al. [19] found that market incentives have significant impacts on farmer behavioral factors externally, which is parallel with our study. In a study on the Ethiopian central rift valley, Mengistie et al. [85] found that farmers’ livelihood expectancy and the daily circumstance impacted the intensity of pesticide applications, which is supported by our study. Interestingly, farmers with higher planting areas and experience have sowed a greater acceptance of conventional pesticides, which is supported by Schreinemachers et al. [100]. Though some studies such as a study on Nigerian cocoa farmers in Tijani [101] and a study on Thai Tangerine farmers, Chalermphol and Shivakoti [102] found different outcomes. It can be seen from the above discussion that market incentives are an important external driving force affecting farmers’ pesticide application behavior, and livelihood dependence is an important internal force affecting farmers’ pesticide application behavior.

## 6. Conclusions

Based on the data of 766 farmer household surveys in key tea areas in Shaanxi, Sichuan, Zhejiang, and Anhui provinces, we empirically analyzed the impact of market incentives and livelihood dependence on farmers’ multi-stage pesticide application behavior. The study found that first, market incentives significantly prompted some farmers to give up pesticide application, but also created a tendency for farmers to choose conventional pesticides in terms of pesticide application types; market incentives had no significant impact on farmers’ conventional pesticide application rates but prompted farmers to reduce green low levels. Second, in the livelihood dependence, the proportion of tea income significantly prompts farmers to apply pesticides, and also creates the tendency for farmers to choose green and low-toxic pesticides in the type of pesticide application, but it has no significant impact on the application rates of farmers with conventional pesticides and green and low-toxic pesticides. The planting year has no significant effect on whether farmers apply pesticides, but it creates the tendency for farmers to choose conventional pesticides in the type of pesticide application; the planting year makes the farmers choose to increase the number of conventional pesticides, and has no significant impact on the amount of green and low-toxic pesticides for farmers. Third, the interaction term of market incentives and the proportion of tea income is not significant; the interaction term of market incentives and planting years prompts farmers to choose pesticides, which has no significant impact on the types of pesticides applied by farmers but makes farmers increase the amount of green and low-toxic pesticides.

Based on the above conclusions, the following implications are drawn. First, strengthen market incentives to ensure the effective transmission of market signals of high quality and good prices. The government should give financial support to market organizations, and provide policy financing for the purchase of necessary inspection equipment, technology introduction fees, etc., or preferential policies such as tax deductions, to improve the ability of market organizations to identify product toxicity; build an agricultural product information data platform to improve the quality of agricultural products. The traceability system enhances the transparency of agricultural product quality information and ensures the effectiveness of market incentives. Second, it provides economic incentives for high-standard pesticide application farmers, strengthens technical training, and implements the upgrading and transformation of old tea gardens and special training projects for old tea farmers. On the one hand, the government should set up a special fund to provide physical or financial support to farmers who use green and low-toxic pesticides and reduce the number of pesticide applications, to reduce farmers’ operating costs and potential risks. On the other hand, the government should increase the knowledge and skill training on pesticide application for farmers through technical training, farmer field schools, etc., to weaken the misunderstanding of planting years, effectively improve farmers’ pesticide skills, and design special projects to upgrade old tea gardens. This gradually reverses the irrational pesticide application behavior of farmers. Third, it formulates differentiated guidance strategies based on fully grasping the relationship between market incentives and livelihood dependence and its impact on farmers’ pesticide application behavior, such as establishing a market credit system, issuing product quality awards, or honorable household awards, financial subsidies, and technical training, to create conditions for farmers with different livelihoods to adapt to market incentives, adapt to large market demands, and green transformation.

In the future, this study can be expanded within the aspects of the connotation and types of market incentives, include more psychological and government regulation variables and build a more realistic decision-making scenario. Moreover, potential studies should focus on deeply revealing the impact of market incentives and livelihood dependence on farmers’ multi-stage pesticide application behavior. The study only focussed on four provinces while future studies can expand their target study area to grasp the whole scenario of the country. The study only explored limited control variables. However, the potential studies should include several crucial control variables which may have certain impacts such as the degree of adopting environmentally friendly pest management tactics. Moreover, the descriptive statistics should be explored more critically and comprehensively according to the local conditions and socio-economical circumstances. 

## Figures and Tables

**Table 1:** Variables and their descriptive statistics.

Variable		Variable Meaning	Mean	Std.
**Dependent variable**				
Whether to spray		Yes = 1; No = 0	0.841	0.366
Choice of spray yype		Conventional pesticides = 0; Green low-toxicity pesticides = 1	0.669	0.471
Choice of dosage	Conventional pesticide dosage	Decrease = 1; Unchanged = 2; Increase = 3	2.211	0.732
	Dosage of green and ow-toxic pesticides	Decrease = 1; Unchanged = 2; Increase = 3	2.155	0.796
**Core variable**				
Market incentives		Yes = 1; No = 0	0.305	0.461
Subsistence dependence	Proportion of tea revenue	Tea revenue/Total revenue	0.255	0.250
	Planting years	Tea planting time as of 2017 (year)	25.372	12.654
**Control variable**				
Head of household characteristics	Age	Actual age (years)	57.832	9.693
	Educational level	Actual cultural level	6.128	3.461
Family characteristics	Labor force	Number of labor force (person)	2.189	0.851
	Family income level	(10,000 Yuan)	6.342	8.970
Field endowment	Tea garden area	Actual planting area (Mu)	7.194	21.639
	Garden elevation	(100 m)	4.342	2.401
	Road condition	Good traffic conditions = 0; Poor traffic conditions = 1	0.918	0.275
Pesticide awareness	Pesticide yield effect	Will the reduction in pesticides lead to a reduction in production? Below 10% = 1; 10–20% = 2; 20–30% = 3; 30–40% = 4; 40–50% = 5; Above 60 = 6	3.097	1.862
	Pesticide brand effect	Will pesticide use damage the brand of origin? Strongly Disagree = 1; Somewhat Disagree = 2; Moderately = 3; Somewhat Agree = 4; Strongly Agree = 5	3.110	0.981
	Environmental effects of pesticides	Will pesticides cause soil and water pollution? Strongly Disagree = 1; Somewhat Disagree = 2; Moderately = 3; Somewhat Agree = 4; Strongly Agree = 5	2.257	1.006
External support	Technical training	Yes = 1; No = 0	0.202	0.402
Area	Shaanxi	Shaanxi = 1; Other = 0	0.360	0.480
	Zhejiang	Zhejiang = 1; Other = 0	0.154	0.361
	Anhui	Anhui = 1; Others = 0	0.232	0.423

**Table 2:** Estimated results and test of whether farmers apply pesticides.

Variable		Model 1		Model 2	
		Cof.	Std.	Cof.	Std.
Core variable					
Market incentives		−0.271 **	0.135	−0.302 **	0.139
Subsistence dependence	Proportion of tea revenue	0.568 *	0.316	0.614 **	0.329
	Planting years	0.077	0.099	0.067	0.099
Market incentives × livelihood dependence	Market incentive × proportion of tea income	-	-	−0.084	0.062
	Market incentive × planting years	-	-	0.104 **	0.048
Control variable					
Head of household characteristics	Age of head of household	0.209	0.367	0.245	0.368
	Education level of the head of the household	0.040 **	0.019	0.042 **	0.019
Family characteristics	Labor force	0.037	0.071	0.038	0.071
	Family income level	−0.037	0.113	−0.033	0.116
Field endowment	Tea garden area	0.033	0.093	0.038	0.092
	Tea garden elevation	−0.084 *	0.049	−0.091 *	0.049
	Road condition	0.200	0.204	0.165	0.210
Effect cognition	Pesticide yield effect	0.219 ***	0.039	0.215 ***	0.039
	Pesticide brand effect	−0.153 **	0.061	−0.151 **	0.062
	Environmental effects of pesticides	0.128	0.080	0.130 *	0.087
External support	Technical training	−0.061	0.149	−0.051	0.149
Regional variable		Yes		Yes	
Sample size		766		766	
Wald chi^2^		75.69		82.25	
Prob > chi^2^		0.000		0.000	

Note: ***, **, and * represent the significant levels of 1%, 5%, and 10%, respectively; the household income level and the area of tea gardens are the logarithms of their actual values.

**Table 3:** Estimated results and tests of farmers’ choice of pesticide application types.

Variable		Model 3		Model 4	
		Cof.	Std.	Cof.	Std.
**Core independent variable**					
Market incentives		−0.348 ***	0.129	−0.389 ***	0.131
Subsistence dependence	Proportion of tea revenue	0.562 **	0.283	0.597 **	0.284
	Planting years	−0.277 ***	0.097	−0.283 ***	0.097
Market incentives × livelihood dependence	Market incentive × proportion of tea income	-	-	−0.039	0.058
	Market incentive × planting years	-	-	−0.339	0.323
**Control variable**					
Head of household characteristics	Age of head of household	−0.367	0.359	−0.344	0.360
	Education level of the head of the household	0.030 *	0.018	0.030 *	0.018
Family characteristics	Labor force	−0.022	0.064	−0.020	0.064
	Family income level	0.072	0.106	0.094	0.107
Field endowment	Tea garden area	−0.073	0.093	−0.082	0.093
	Tea garden elevation	0.062	0.047	0.063	0.047
	Road condition	0.252	0.205	0.252	0.204
Effect cognition	Pesticide yield effect	0.057 *	0.031	0.056 *	0.031
	Pesticide brand effect	0.264 ***	0.059	0.264 ***	0.059
	Environmental effects of pesticides	−0.102	0.071	−0.095	0.061
External support	Technical training	0.516 ***	0.156	0.504 ***	0.156
Regional variable		Yes		Yes	
Sample size		644		644	
Wald chi^2^		98.62		99.11	
Prob		0.000		0.000	

Note: ***, **, and * represent the significant levels of 1%, 5%, and 10%, respectively; the household income level and the area of tea gardens are the logarithms of their actual values.

**Table 4:** Estimated results and test of farmers’ choice of pesticide application rate.

Variable		Model 5		Model 6		Model 7		Model 8	
		Conventional Pesticide Dosage				Dosage of Green and Low-Toxic Pesticides			
		Cof.	Std.	Cof.	Std.	Cof.	Std.	Cof.	Std.
**Core independent variable**									
Market incentives		−0.038	0.174	0.094	0.183	−0.313 **	0.150	−0.237	0.154
Subsistence dependence	Proportion of tea revenue	−0.518	0.490	−0.405	0.488	0.056	0.259	0.008	0.259
	Planting years	0.490 ***	0.133	0.489 ***	0.133	0.116	0.108	0.136	0.109
Market incentives × livelihood dependence	Market incentive × proportion of tea income	-	-	−0.091	0.104	-	-	−0.017	0.059
	Market incentive × planting years	-	-	−0.309	0.483	-	-	0.802 **	0.367
**Control variable**									
Head of household characteristics	Age of head of household	−0.057	0.516	−0.005	0.516	0.070	0.367	0.046	0.368
	Education level of the head of the household	−0.018	0.026	−0.019	0.025	0.028	0.019	0.025	0.019
Family characteristics	Labor force	0.023	0.100	0.034	0.100	0.071	0.070	0.065	0.069
	Family income level	0.036	0.162	0.057	0.163	0.234 **	0.109	0.213 *	0.112
Field endowment	Tea garden area	−0.231 *	0.132	−0.243 *	0.145	−0.221 **	0.103	−0.208 **	0.104
	Tea garden elevation	0.011	0.061	0.013	0.060	−0.058	0.054	−0.065	0.054
	Road condition	−0.512 *	0.271	−0.517 *	0.270	−0.208	0.239	−0.178	0.240
Pesticide awareness	Pesticide yield effect	0.095 **	0.048	0.088 *	0.048	0.050	0.034	0.050	0.034
	Pesticide brand effect	−0.252 **	0.101	−0.243 **	0.103	−0.152 **	0.060	−0.148 **	0.060
	Environmental effects of pesticides	−0.105	0.089	−0.085	0.090	−0.078	0.066	−0.084	0.065
External support	Technical training	−0.555 **	0.243	−0.617 **	0.251	−0.047	0.151	−0.057	0.152
Regional variable		Yes		Yes		Yes		Yes	
Wald chi^2^		55.86		54.19		43.67		46.67	
Prob		0.000		0.000		0.000		0.000	
Sample size		213		213		431		431	

Note: ***, **, and * represent the significant levels of 1%, 5%, and 10%, respectively; the household income level and the area of tea gardens are the logarithms of their actual values.

**Table 5:** Robustness test regression results.

Variable	Model 9		Model 10		Model 11		Model 12		Model 13		Model 14		Model 15		Model 16	
	Whether to Spray				Type of Application				Conventional Pesticide Dosage				Dosage of Green and Low-Toxic Pesticides			
	Cof.	Std.	Cof.	Std.	Cof.	Std.	Cof.	Std.	Cof.	Std.	Cof.	Std.	Cof.	Std.	Cof.	Std.
Market Incentives	−0.444 *	0.245	−0.520 **	0.256	−0.586 ***	0.214	−0.658 ***	0.216	−0.079	0.297	−0.206	0.323	−0.510 **	0.256	−0.390	0.264
Proportion of Tea Revenue	0.911 *	0.508	1.032 *	0.602	0.945 **	0.482	1.019 **	0.485	−0.791	0.854	−0.622	0.831	0.033	0.424	−0.029	0.429
Planting Years	0.125	0.180	0.107	0.179	−0.464 ***	0.166	−0.471 ***	0.165	0.831 ***	0.229	0.831 ***	0.232	0.215	0.187	0.251	0.190
Market Incentive × Proportion of Tea Income	-	-	−0.153	0.115	-	-	−0.081	0.097	-	-	−0.200	0.197	-	-	−0.027	0.098
Market Incentive × Planting Years	-	-	0.164 **	0.083	-	-	−0.551	0.539	-	-	−0.597	0.848	-	-	1.295 **	0.631
Control Variable	Yes		Yes		Yes		Yes		Yes		Yes		Yes		Yes	
Sample Size	766		766		644		644		213		213		431		431	
Wald Chi^2^	71.85		77.10		93.01		92.74		50.95		48.10		41.63		48.79	
Prob	0.000		0.000		0.000		0.000		0.000		0.000		0.000		0.000	

Note: The control variables have been controlled and are not shown in detail due to space limitations. ***, **, and * represent the significant levels of 1%, 5%, and 10%, respectively; the household income level and the area of tea gardens are the logarithms of their actual values.

## Data Availability

The associated dataset for the study is available upon request to the corresponding author.
